# m6A Regulators Is Differently Expressed and Correlated With Immune Response of Esophageal Cancer

**DOI:** 10.3389/fcell.2021.650023

**Published:** 2021-03-04

**Authors:** Huaying Zhao, Yue Xu, Yilin Xie, Lan Zhang, Ming Gao, Shenglei Li, Feng Wang

**Affiliations:** ^1^Department of Pathology, The First Affiliated Hospital, Zhengzhou University, Zhengzhou, China; ^2^Department of Oncology, The First Affiliated Hospital of Zhengzhou University, Zhengzhou, China

**Keywords:** m6A, RNA methylation, esophageal cancer, TCGA, immune infiltration

## Abstract

N6 methyladenosine (m6A) RNA methylation regulators play an important role in the development of tumors. However, their function in esophageal cancer (EC) has not been fully elucidated. Here, we analyzed the gene expression data of 24 major m6A RNA methylation regulators from 775 patients with EC from TCGA dataset. The present study showed the aberrations of m6A regulators in genome were correlated to prognosis in human ECs. Meanwhile, 17 m6A regulators showed increased expression in EC samples, including YTHDC1, IGF2BP2, FTO, METTL14, YTHDF3, RBM15, WTAP, HNRNPA2B1, HNRNPC, ALKBH5, YTHDF2, METTL16, IGF2BP3, VIRMA, RBM15B, YTHDF1, KIAA1429, HAKAI, and ZC3H13. Among them, we found HNRNPC, YTHDC2, WTAP, VIRMA, IGF2BP3, and HNRNPA2B1 were significantly correlated to worse outcomes and advanced stage in EC. Furthermore, we showed levels of m6A regulators is correlated with the expression of Immuno-regulators (Immunoinhibitors, Immunostimulators, and MHC molecules) and immune infiltration levels in EC. Bioinformatics further confirm m6A regulators were involved in regulating RNA splicing, RNA stability, and cell proliferation. Our study showed m6A regulators are promising targets and biomarkers for cancer immunotherapy in EC.

## Introduction

Esophageal cancer (EC) accounts for 1% of all cancer cases, had been the eighth most commonly diagnosed cancer (Malhotra et al., 2017; Fan et al., 2020). According to the anatomic location of the tumor, esophageal carcinoma can be divided into esophageal adenocarcinoma and esophageal squamous cell carcinoma (ESCC) (Salem et al., 2018). ESCC is the main subtype of EC in developing countries, accounting for more than 90% of all subtypes of EC in China (Liang et al., 2017). Esophagectomy is the standard treatment for EC. Radiotherapy is an effective treatment option to cure or control EC (Jin et al., 2017). Targeted therapy is a key step in the development of individualized treatment for EC. EC is one of the most immune-infiltrated tumors. Signals in immune microenvironment, including accumulation of tumor metabolites or T cell dysfunction, may significantly affect the response to immune checkpoint therapy (ICT) in EC patients (Wu et al., 2020). In recent years, the development of monoclonal antibodies against programmed death 1 (PD-1) or programmed death ligand 1 (PD-L1) has achieved convincing efficacy and clinical benefits in a variety of malignant tumors including ESCC (Yuan et al., 2017; Baba et al., 2020).

m6A modification in RNA is a dynamic and reversible process, which is related to many diseases, such as cancer (Yue et al., 2015; He et al., 2017; Fan et al., 2019; Gu et al., 2020b). Recently, several progresses had been made in RNA splicing, stability and Translation regulation through the post-transcriptional modification of m6A (Zhang et al., 2019a). New evidences showed that m6A modification is associated with tumor proliferation, glycolysis, apoptosis, and metastasis (Dai et al., 2020). m6A modification could act as either an oncogenetic role or tumor suppressive role in malignant tumors. Studies have shown that m6A mRNA methylation modification is reversible and is dynamically regulated by methyltransferases that include METTL3/14, WTAP, and KIAA1429 (Bi et al., 2019). Meanwhile, several other RNA binding proteins were also reported to modulate m6A progression, such as HNRNPC, YTHDC2, WTAP, VIRMA, IGF2BP3, and HNRNPA2B1. These regulators had been demonstrated to have a crucial role in cancers. For example, HNRNPC facilitates progression of oral squamous cell carcinoma via EMT (Huang et al., 2020). WTAP could enhance the Warburg effect of gastric cancer through regulating HK2 stability (Yu et al., 2021). Although the m6A modification can affect the tumorigenesis in a variety of tumors, the mechanism of m6A in EC is still unclear.

This study used the data in the Tumor Genome Atlas (TCGA) database to analyze the expression of m6A methylation regulator in EC and its relationship with clinicopathological characteristics and we also used bioinformatics methods to predict the potential functions of these m6A regulators. The analysis showed that the m6A regulatory factor may be a potential immunotherapy target and biomarker for EC.

## Materials and Methods

### Expression Analysis

Gepia 2^1^ was used to compare the expression of m6A regulators (Tang et al., 2017). UALCAN was used to confirmed the correlation between m6A regulators and clinical parameters in EC (Chandrashekar et al., 2017).

### Immune Response Prediction

In this study, we detected the correlation of m6A regulators with levels of immune cell infiltration (including Cancer associated fibroblast, Myeloid dendritic cell, CD4+ T cell, Neutrophil, T cell regulatory (Tregs), CD8+ T cell, Macrophage) in EC using the TIMER database (Li et al., 2017).

the correlations between the expression of m6A regulators and Immuno regulators (including Immunoinhibitors, Immunostimulator, and MHC molecules) were calculated using TISIDB database^2^ (Ru et al., 2019).

#### Protein-Protein Interaction PPI Networks and Hub Genes

A PPI network was constructed based on DEGs using the STRING database and visualized by the Cytoscape software (Gu et al., 2020c). The cut-off value was defined as an interaction score (median confidence) of 0.4.

#### Genetic Alteration of m6A Regulators in EC

CBioPortal^3^ is an open-access website that explores, visualizes, and analyzes multidimensional cancer genomics data, which was used to analyze the genetic alterations of m6A regulators in EC.

### Survival Analysis

The correlation between m6A regulators aberrations and survival time in human cancers was determined using cBioPortal database (Unberath et al., 2019). The correlation between overalls survival time and m6A regulators expression are measured using KM diagrams and are determined by the previously reported endpoints^4^ (Gyorffy et al., 2013; Gu et al., 2020a).

### GO and KEGG Analysis

Using The Database for Annotation, Visualization, and Integrated Discovery (DAVID)^5^, we performed Gene ontology (GO) and Kyoto Encyclopedia of Genes and Genomes (KEGG) pathway enrichment analysis based on the co-expression genes. and a critical value of *P* << 0.05 is selected as cutoff (Shi et al., 2018a,b).

### Statistical Data

For all the above analyses, except those specifically mentioned, a *P*-value less than 0.05 was regarded as statistically significant.

## Results

### The Aberrations of m6A Regulators in Genome Were Correlated to Prognosis in Human Cancers

**FIGURE 1:**
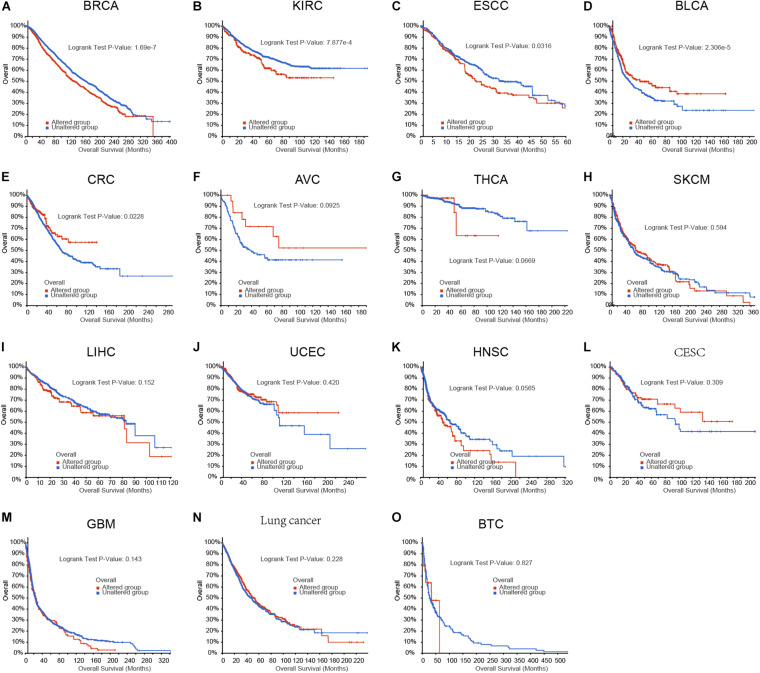
The aberrations of m6A regulators in genome were correlated to prognosis in human cancers. **(A–O)** The correlation between m6A regulators aberrations and overall survival time in patient with breast cancer, kidney cancer, EC, bladder cancer, colon cancer, ampulla of vater cancer (AVC), Thyroid cancer, skin cancer, liver cancer, uterus cancer, head and neck cancer, cervix cancer, glioblastoma, lung cancer, biliary cancer.

To evaluate the functional importance of m6A regulators in human cancers, we analyzed the correlation between m6A regulators aberrations and survival time in human cancers using cBioPortal database. As present in Figure 1, we observed the aberrations of m6A regulators in genome were remarkably correlated to worse prognosis in patients with breast cancer, kidney cancer and EC, however, were remarkably correlated to longer OS time in patients with bladder cancer, and colon cancer. In addition, we did not find a significantly correlation between m6A regulators genomic aberration and survival time in other types of human cancers.

### Amplification, Deletion, and Mutation of m6A Regulators in EC

**FIGURE 2:**
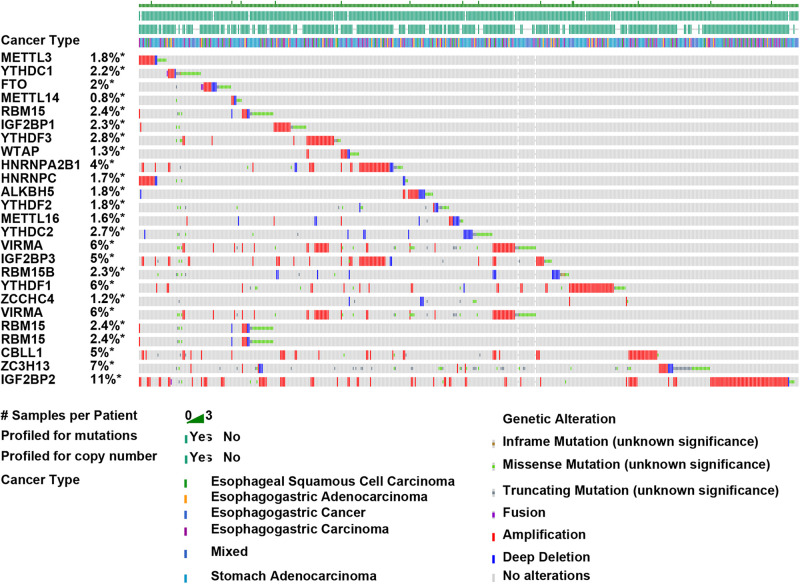
Amplification, deletion, and mutation of m6A regulators in EC. Genetic variations of m6A regulators in 1,680 cases were detected using the cBioPortal database.

The roles of m6A regulators in breast cancer and kidney cancer had been implied in previous studies. The present study focused on exploring the roles of m6A regulators in EC. Genetic variations of m6A regulators in 1,680 cases were detected using the cBioPortal database (Figure 2). We found varying degrees of genetic changes among the 23 m6A regulators, including, ALKBH5, FTO, HAKAI, HNRNPA2B1, HNRNPC, IGF2BP1, IGF2BP2, IGF2BP3, KIAA1429, METTL14, METTL16, METTL3, RBM15, RBM15B, VIRMA, WTAP, YTHDC1, YTHDC2, YTHDF1, YTHDF2, YTHDF3, ZC3H13, ZCCHC4. As present in Figure 2, we revealed most of m6A regulators were amplified, deleted, mutated in EC, among which IGF2BP2displayed the highest incidence rate (11%).

### Expression Profile of m6A Regulators in EC

**FIGURE 3:**
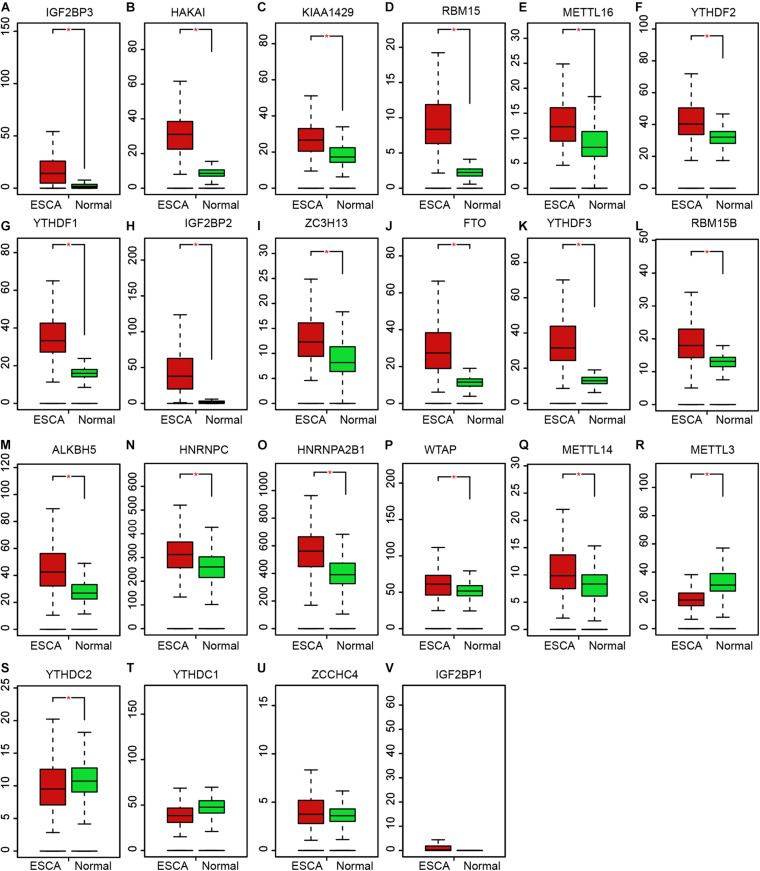
Expression profile of m6A regulators in EC. **(A–V)** The expression levels of m6A regulators showed increased expression in EC samples, including IGF2BP3 **(A)**, HAKAI **(B)**, KIAA1429 **(C)**, RBM15 **(D)**, METTL16 **(E)**, YTHDF2 **(F)**, YTHDF1 **(G)**, IGF2BP2 **(H)**, ZC3H13 **(I)**, FTO **(J)**, YTHDF3 **(K)**, RBM15B **(L)**, ALKBH5 **(M)**, HNRNPC **(N)**, HNRNPA2B1 **(O)**, WTAP **(P)**, METTL14 **(Q)**, METTL3 **(R)**, and YTHDC2 **(S)**, YTHDC1 **(T)**, ZCCHC4 **(U)**, IGF2BP1 **(V)**. *P < 0.05 compared with normal tissues.

The level of m6A regulators in TCGA was presented in the Figure 3. Among which, 17 m6A regulators showed increased expression in EC samples, including IGF2BP3 (Figure 3A), HAKAI (Figure 3B), KIAA1429 (Figure 3C), RBM15 (Figure 3D), METTL16 (Figure 3E), YTHDF2 (Figure 3F), YTHDF1 (Figure 3G), IGF2BP2 (Figure 3H), ZC3H13 (Figure 3I), FTO (Figure 3J), YTHDF3 (Figure 3K), RBM15B (Figure 3L), ALKBH5 (Figure 3M), HNRNPC (Figure 3N), HNRNPA2B1 (Figure 3O), WTAP (Figure 3P), METTL14 (Figure 3Q). Meanwhile, METTL3 (Figure 3R), and YTHDC2 (Figure 3S) were suppressed in tumor samples. However, we found YTHDC1 (Figure 3T), ZCCHC4 (Figure 3U), IGF2BP1 (Figure 3V) were not differently expressed between normal and tumor samples.

### The Dysregulation of m6A Regulators Were Correlated to Shorter OS Time in EC

**FIGURE 4:**
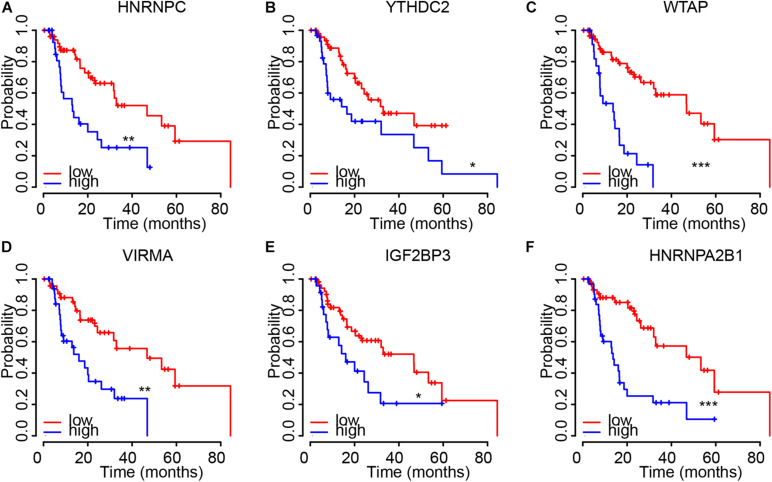
The dysregulation of m6A regulators were correlated to shorter OS time in EC. **(A–F)** higher levels of HNRNPC **(A)**, YTHDC2 **(B)**, WTAP **(C)**, VIRMA **(D)**, IGF2BP3 **(E)**, and HNRNPA2B1 **(F)** were significantly associated with worse outcomes in EC. *p < 0.05; **p < 0.01; ***p < 0.0001.

Then, Kaplan–Meier plot was used to detect the prognostic value of m6A regulators in EC. We revealed higher levels of HNRNPC (Figure 4A), YTHDC2 (Figure 4B), WTAP (Figure 4C), VIRMA (Figure 4D), IGF2BP3 (Figure 4E), and HNRNPA2B1 (Figure 4F) were significantly associated with worse outcomes in EC, indicating these m6A regulators had key roles in EC.

### The Dysregulation of m6A Regulators Were Correlated to Advanced Clinical Stage in EC

**FIGURE 5:**
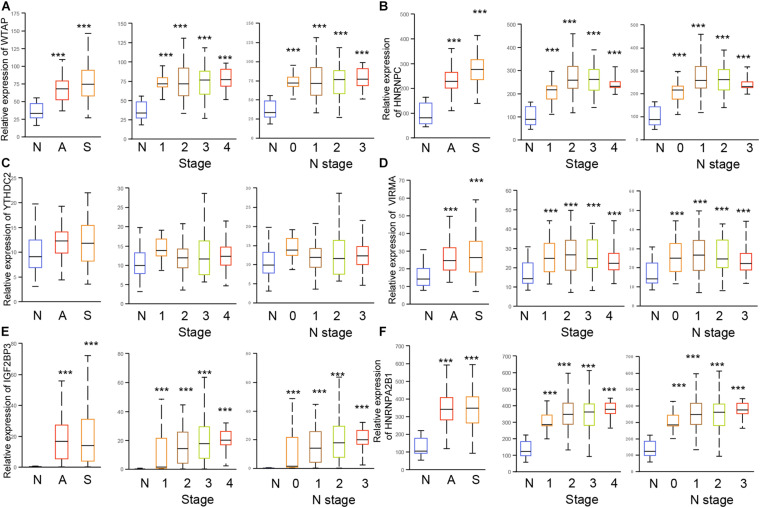
The dysregulation of m6A regulators were correlated to advanced cinical stage in EC. **(A–F)** The expression of HNRNPC, YTHDC2, WTAP, VIRMA, IGF2BP3, and HNRNPA2B1 were detected in esophageal squamous cell carcinoma, esophageal adenocarcinoma, and stage 1/2/3/4, N 0/1/2/3. N: normal samples; A: esophageal adenocarcinoma; S: Esophageal squamous cell carcinoma. ***p < 0.0001.

We next confirmed the correlation between m6A regulators and clinical parameters in EC. As showed in Figure 4, the results showed all of 6 m6A regulators (HNRNPC, YTHDC2, WTAP, VIRMA, IGF2BP3, and HNRNPA2B1) were up-regulated in both Esophageal squamous cell carcinoma and esophageal adenocarcinoma compared to normal samples. Among these genes, IGF2BP3 showed the most significantly up-regulation in EC samples compared to normal tissues (Figure 5E). In addition, we found only WTAP (Figure 5A) and HNRNPC (Figure 5B) were up-regulated in squamous cell carcinoma compared to adenocarcinoma.

The further analysis indicated that WTAP (Figure 5A), HNRNPC (Figure 5B), YTHDC2 (Figure 5C), VIRMA (Figure 5D), IGF2BP3 (Figure 5E), HNRNPA2B1 (Figure 5F) were up-regulated in all clinical stages and N stages of EC. Meanwhile, HNRNPC (Figure 5B), IGF2BP3 (Figure 5E), and HNRNPA2B1 (Figure 5F) were up-regulated in stage 2/3/4 samples compared to stage 1 sample, and up-regulated in N1/2/3 samples compared to N0 samples. YTHDC2 (Figure 5C) was revealed to be down-regulated in stage 2/3/4 samples compared to stage 1 sample, and down-regulated in N1/2/3 samples compared to N0 samples. WTAP (Figure 5A), VIRMA (Figure 5D) were not differently expressed among different stages of EC.

### m6A Regulators Expression Is Correlated With Immune Infiltration Levels in EC

**FIGURE 6:**
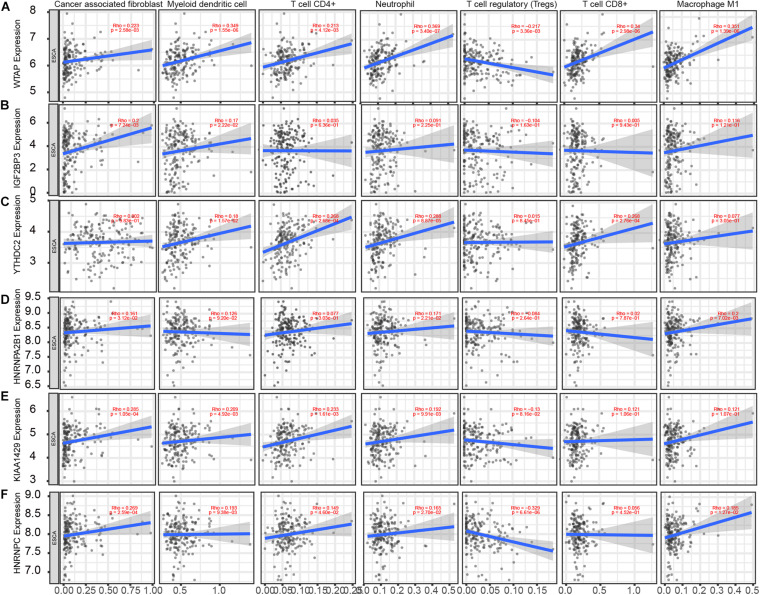
m6A regulators expression is correlated with immune infiltration levels in EC. **(A–F)** Based on the TIMER database, we detected the correlation of WTAP **(A)**, IGF2BP3 **(B)**, YTHDC2 **(C)**, HNRNPA2B1 **(D)**, VIRMA **(E)**, HNRNPC **(F)** with levels of Cancer associated fibroblast, Myeloid dendritic cell, CD4+ T cell, Neutrophil, T cell regulatory (Tregs), CD8+ T cell, Macrophage.

Based on the TIMER database, we detected the correlation of m6A regulators with levels of immune cell infiltration in EC. As present in Figure 6, WTAP (Figure 6A) was associated with Cancer associated fibroblast (*r* = 0.223, *p* = 2.58e–03), Myeloid dendritic cell (*r* = 0.349, *p* = 1.55e–06), CD4+ T cell (*r* = 0.213, *p* = 4.12e–03), Neutrophil (*r* = 0.369, *p* = 3.40e–07), T cell regulatory (Tregs) (*r* = −0.217, *p* = 3.36e–03), CD8+ T cell (*r* = 0.34, *p* = 2.98e–06), Macrophage (*r* = 0.351, *p* = 1.39e–06). IGF2BP3 (Figure 6B) was significantly associated with Cancer associated fibroblast (*r* = 0.2, *p* = 7.24e–03), Myeloid dendritic cell (*r* = 0.17, *p* = 2.22e–02). YTHDC2 (Figure 6C) was significantly associated with CD4+ T cell (*r* = 0.268, *p* = 2.68e–04), Neutrophil (*r* = 0.288, *p* = 8.87e–05), CD8+ T cell (*r* = 0.268, *p* = 2.76e–04). HNRNPA2B1 (Figure 6D) was significantly associated with Macrophage (*r* = 0.2, *p* = 7.02e–03). VIRMA (Figure 6E) was significantly associated with Cancer associated fibroblast (*r* = 0.285, *p* = 1.05e–04), Myeloid dendritic cell (*r* = 0.209, *p* = 4.92e–03), CD4+ T cell (*r* = 0.233, *p* = 1.61e–03), Neutrophil (*r* = 0.192, *p* = 9.91e–03). HNRNPC (Figure 6F) was significantly associated with Cancer associated fibroblast (*r* = 0.269, *p* = 2.59e–04), Myeloid dendritic cell (*r* = 0.193, *p* = 9.38e–03), Neutrophil (*r* = −0.329, *p* = 6.61e–06).

### Correlations Between m6A Regulators Expression and the Expression of Immuno Regulators in EC

**FIGURE 7:**
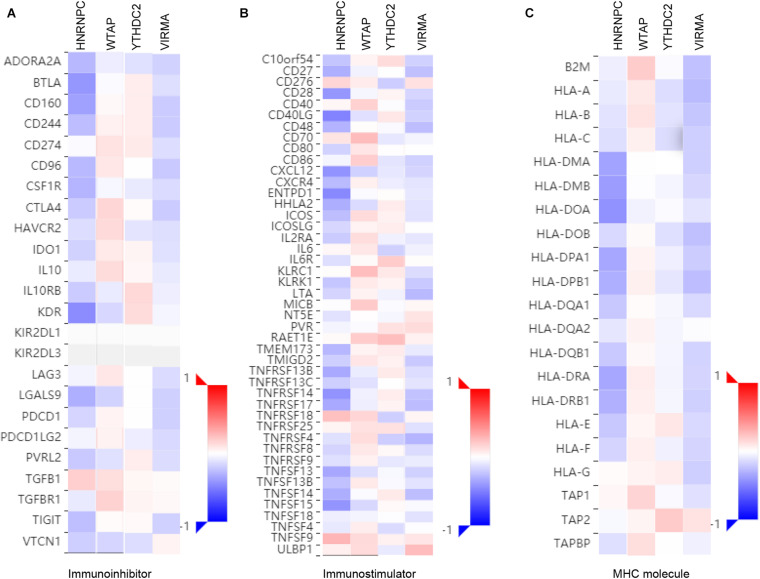
Correlations between m6A regulators Expression and the expression of Immuno regulators in EC. The correlations between the expression of HNRNPC, VIRMA, WTAP, andYTHDC2 and Immunoinhibitors **(A)**, Immunostimulator **(B)**, and MHC molecules **(C)** were calculated based on TISIDB database.

To further explore the effects of m6A regulators on tumor immune response, the correlations between the expression of m6A regulators and Immuno regulators were calculated. The results indicated that HNRNPC and VIRMA were negatively correlated to Immunoinhibitors (Figure 7A), Immunostimulators (Figure 7B), and MHC molecules (Figure 7C). However, we found WTAP were positively correlated to Immunoinhibitors, Immunostimulator, and MHC molecules in EC (Figure 7). In addition, YTHDC2 level was positively related to the expression levels of Immunoinhibitors, Immunostimulator, however, was negatively correlated to MHC molecules’ expression in EC (Figure 7).

### GO and KEGG Enrichments

**FIGURE 8:**
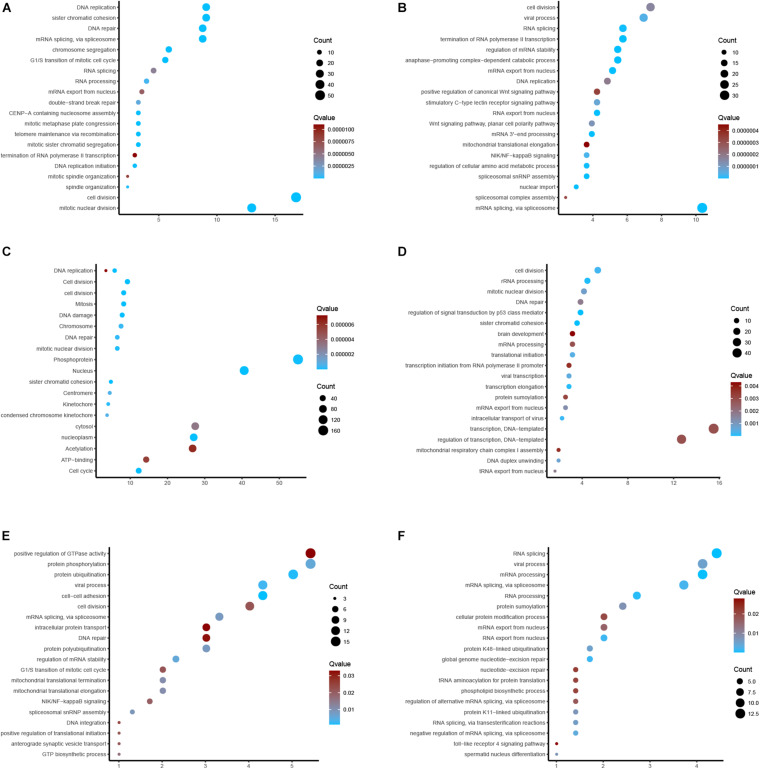
GO and KEGG enrichments. **(A–F)** Bioinformatics analysis of YTHDC2, Wtap, VIRMA, IGF2BP3, HNRNPC, HNRNPA2B1 in EC.

For the sake of investigating the downstream pathways of hub m6A regulators in EC, we performed GO and KEGG analysis using co-expression genes of 6 m6A regulators. The results showed that YTHDC2 was related to cell-cell adhesion, protein ubiquitination, viral process, regulation of mRNA stability, protein phosphorylation, mRNA splicing, via spliceosome, protein polyubiquitination, spliceosomal snRNP assembly, mitochondrial translational elongation, mitochondrial translational termination, GTP biosynthetic process, NIK/NF-kappaB signaling, G1/S transition, translational initiation, anterograde synaptic vesicle transport, cell division, DNA integration, DNA repair, intracellular protein transport (Figure 8A). Wtap was related to RNA splicing, mRNA processing, RNA processing, global genome nucleotide-excision repair, RNA export from nucleus, mRNA splicing, viral process, protein K48-linked ubiquitination, RNA splicing, via transesterification reactions, protein K11-linked ubiquitination, spermatid nucleus differentiation, protein sumoylation (Figure 8B). VIRMA was related to regulation of p53 signal, sister chromatid cohesion, rRNA processing, transcription elongation, intracellular transport of virus, cell division, translational initiation, viral transcription, DNA duplex unwinding, mitotic nuclear division, mRNA export from nucleus, tRNA export from nucleus, DNA repair (Figure 8C). IGF2BP3 was related to Mitosis, Cell cycle, Cell division, DNA replication, nucleoplasm, Nucleus, DNA damage, cell division, Phosphoprotein, Kinetochore, DNA repair (Figure 8D). HNRNPC was related to mRNA splicing, spliceosomal snRNP assembly, regulation of mRNA stability, nuclear import, RNA export from nucleus, RNA splicing, NIK/NF-kappaB signaling, Wnt signaling, cell division, DNA replication (Figure 8E). HNRNPA2B1 was related to cell division, mitotic nuclear division, mRNA splicing, via spliceosome, DNA repair, G1/S transition, mitotic sister chromatid segregation, telomere maintenance via recombination, mitotic metaphase plate congression, CENP-A containing nucleosome assembly, DNA replication initiation, spindle organization, and RNA processing (Figure 8F).

## Discussion

Over the past decades, the roles of m6A regulators in EC had been revealed in several previous studies. For example, METTL3 enhanced the progression of EC cells through the Akt signaling pathway (Hou et al., 2020). The up-regulation of METTL3 expression indicates a poor prognosis in patients with ESCC. RBM15 had been reported to be dysregulated in ESCC (Xu et al., 2020). The high expression of IGF2BP2 is related to the short-term survival and metastasis of EC (Xu et al., 2020). The m6A reader hnRNPA2B1 is reported to promote the progression of EC by up-regulating ACLY and ACC1 (Guo et al., 2020). The RNA-binding protein heterologous ribonucleoprotein C (HNRNPC) simultaneously interacts with LBX2-AS1, ZEB1, and ZEB2 (Zhang et al., 2019b; Wang et al., 2020). It is confirmed that HNRNPC has similar functions in regulating migration and EMT (Huang et al., 2020). IGF2BP3 is a radioresistance factor in squamous esophageal cancer cells. In this study, we observed the aberrations of m6A regulators in genome were remarkably correlated to worse prognosis in patients with breast cancer, kidney cancer and EC. The roles of m6A regulators in bladder cancer and colon cancer had been implied in previous studies. For example, Chen et al. (2019a) reported m6A RNA methylation regulators can participate in the malignant progression of bladder cancer. However, in EC, there was still lacking of comprehensively analysis of the correlation between m6A regulators and cancer progression. In this study, by using multiple data sets, a significant prognostic value of m6A modulators was observed in EC. In addition, the relationship between m6A regulatory factors and anti-tumor immune response is also investigated. Our research proved for the first time that m6A regulators can be used as potential biomarkers for the prognosis and immunotherapy in EC.

In this study, we found the aberrations of m6A regulators in genome were correlated to prognosis in human ECs. In this study, we revealed most of m6A regulators were amplified, deleted, mutated in EC, among which IGF2BP2 displayed the highest incidence rate (11%). IGF2BP2 is an RNA binding protein, which had a crucial role in m6A modification regulation, mRNA localization, stability and translation. Emerging studies report this protein was related to cancer cell growth and metastasis. For example, Up-regulation of IGF2BP2 promotes pancreatic cancer proliferation by activating the PI3K/Akt signaling pathway (Xu et al., 2019), enhances liver cancer growth through an m6A-FEN1-dependent mechanism. Meanwhile, 17 m6A regulators showed increased expression in EC samples, including ALKBH5, FTO, HAKAI, HNRNPA2B1, HNRNPC, IGF2BP2, IGF2BP3, KIAA1429, METTL14, METTL16, RBM15, RBM15B, VIRMA, WTAP, YTHDF1, YTHDF2, YTHDF3, ZC3H13. Among them, we found HNRNPC, YTHDC2, WTAP, VIRMA, IGF2BP3, and HNRNPA2B1 were significantly correlated to worse outcomes and advanced stage in EC, indicating these m6A regulators play important roles in EC and hold the key to the prognosis of patients. Among these genes, WTAP is the key subunit of the m(6)A methyltransferase complex, and had a crucial role in cancers. For example, WTAP suppressed HMBOX1 expression in an m6A-dependent manner in osteosarcoma tumorigenesis (Chen et al., 2020). Overexpression of WTAP contributed to poor prognosis of gastric cancer by affecting tumor-associated T lymphocyte infiltration (Zhang et al., 2020). In liver cancer, WTAP suppressed ETS1 expression via m6A-HuR-dependent epigenetic silencing (Chen et al., 2019b). IGF-2 mRNA binding proteins, including IGF2BP3, HnRNPA2B1, and HnRNPC, have been identified as m6A readers, which was involved in modulating RNA stability, translation, splicing, decay, and Subcellular localization. hNRNPA2B1 can bind to transcripts containing m6A modification via “m6A-switch” mechanisms (Liu and Pan, 2016). The results showed that HNRNPA2B1 was increased significantly in ESCA and positively associated with ESCA tumor stage and lymph node metastasis. In addition, knocking down hnRNPA2B1 can inhibit ESCA growth and metastasis (Guo et al., 2020). In non-small cell lung cancer, down-regulation of the m6A reader YTHDC2 promotes tumor progression and predicts poor prognosis (Sun et al., 2020). YTHDC2 activates the IGF1R/Akt/S6 signal axis to promote radiotherapy tolerance for nasopharyngeal carcinoma (He et al., 2020). A previous study also showed a single nucleotide polymorphism (SNP) rs2416282 in the YTHDC2 gene promoter region is significantly associated with ESCC susceptibility (Yang et al., 2020). KIAA1429 enhanced liver tumorigenesis through regulating GATA3 in a m6A-dependent manner and also act as an oncogene in breast cancer by modulating CDK1 (Qian et al., 2019).

ICT with anti-PD-1 and anti-PD-L1 therapy has completely changed the treatment of various advanced cancers (Bacot et al., 2020), including EC. Despite immune checkpoint inhibitors (ICI) can significantly improve the prognosis of patients with EC, there are still a considerable proportion of patients who have no response or resistance to ICT. There is increasing evidence that intrinsic factors in tumor cells (e.g., PD-L1 expression, TMB, and MSI-H) are associated with the efficacy of immune checkpoint inhibitors. In addition, external factors including tumor infiltrating lymphocytes (TIL) can also lead to cancer resistance to immunotherapy, tumor associated macrophages (TAM), and myeloid suppressor cells (MDSC). So as to deepen the understanding of tumor immune microenvironment. For example, PD-L1 expression, TIL, TAMs, and MDSCs play an increasingly important role. In present study, we evaluated the correlation between m6A and the level of immune cell infiltration in cancer from TISIDB. It is worth noting that WTAP is associated with cancer-related fibroblasts, myeloid dendritic cells, CD4+ T cells, neutrophil regulatory T cells, CD8+ T cells and macrophages. CD8+ T cells are the key undertakers of anti-tumor immunity, which will further proliferate and differentiate into effective cytotoxic cells with specific cancer killing ability after stimulated by tumor antigens and cytokines secreted by Th1 cells. We also performed bioinformatics analysis of m6A in ESCC. Our results showed that, except m6A and immune response, our results also showed that YTHDC2 was related to cell-cell adhesion, regulation of mRNA stability, NIK/NF-kappaB signaling, G1/S transition of mitotic cell cycle. Wtap was related to RNA splicing. VIRMA was related to p53 signal and sister chromatid cohesion. IGF2BP3 and HNRNPA2B1 were related to multiple proliferation related pathways, including Mitosis, Cell cycle, Cell division, DNA replication. HNRNPC was related to mRNA splicing. Our bioinformatics analysis were consistent with previous reports that these m6A regulators had a key role in regulating cancer cell proliferation. Interestingly, our study also revealed several novel functional roles of these m6A regulators in EC, such as NIK/NF-kappaB signaling. The classical activation pathway of NF-κB signaling has been identified to be related to gut development and repair, innate immunity and inflammation and have a regulatory role in inflammation-associated malignancies. In esophageal adenocarcinoma, elevated NF-κB expression was related to advanced stages and neoadjuvant chemotherapy and radiation response (Gambhir et al., 2015). Our findings indicated that m6A also modulate inflammation-related EC via NF-κB signaling.

Several limitations should be noted in this study. First, TCGA data is used for survival analysis. The validation of mRNA and protein levels in surgical samples from patients with EC further supports the work of m6A modulator as an executable clinical biomarker. Finally, the function of m6A regulatory factor in EC will be further explored using loss-of function assays.

## Conclusion

In conclusion, our study confirmed the dysregulation of tumor associated m6A regulator through bioinformatics analysis, which is associated with prognosis of EC patients, so it can be used as a prognostic biomarker. Furthermore, we showed m6A regulators expression is correlated with immune infiltration levels and the expression of Immuno regulators in EC. Our study indicated m6A regulators may work as a putative drug target in EC.

## Data Availability Statement

All datasets generated for this study are included in the article/supplementary material, further inquiries can be directed to the corresponding author/s.

## Author Contributions

SL and FW conceived and designed the study. HZ, YX, YLX, and LZ performed the analyses. All authors wrote the manuscript, read and approved the manuscript.

## Conflict of Interest

The authors declare that the research was conducted in the absence of any commercial or financial relationships that could be construed as a potential conflict of interest.

## Footnotes

1 http://gepia2.cancer-pku.cn/

2 http://cis.hku.hk/TISIDB

3 http://www.cbioportal.org/

4 http://kmplot.com/

5 http://davidd.ncifcrf.gov/
